# Oligonucleotide Primers for Targeted Amplification of Single-Copy Nuclear Genes in Apocritan Hymenoptera

**DOI:** 10.1371/journal.pone.0039826

**Published:** 2012-06-29

**Authors:** Gerrit Hartig, Ralph S. Peters, Janus Borner, Claudia Etzbauer, Bernhard Misof, Oliver Niehuis

**Affiliations:** 1 Zoologisches Forschungsmuseum Alexander Koenig, Zentrum für Molekulare Biodiversitätsforschung, Bonn, Germany; 2 Universität Münster, Institut für Bioinformatik, Münster, Germany; 3 Zoologisches Forschungsmuseum Alexander Koenig, Abteilung Arthropoda, Bonn, Germany; 4 Universität Hamburg, Biozentrum Grindel und Zoologisches Museum, Hamburg, Germany; Brigham Young University, United States of America

## Abstract

**Background:**

Published nucleotide sequence data from the mega-diverse insect order Hymenoptera (sawflies, bees, wasps, and ants) are taxonomically scattered and still inadequate for reconstructing a well-supported phylogenetic tree for the order. The analysis of comprehensive multiple gene data sets obtained via targeted PCR could provide a cost-effective solution to this problem. However, oligonucleotide primers for PCR amplification of nuclear genes across a wide range of hymenopteran species are still scarce.

**Findings:**

Here we present a suite of degenerate oligonucleotide primer pairs for PCR amplification of 154 single-copy nuclear protein-coding genes from Hymenoptera. These primers were inferred from genome sequence data from nine Hymenoptera (seven species of ants, the honeybee, and the parasitoid wasp *Nasonia vitripennis*). We empirically tested a randomly chosen subset of these primer pairs for amplifying target genes from six Hymenoptera, representing the families Chrysididae, Crabronidae, Gasteruptiidae, Leucospidae, Pompilidae, and Stephanidae. Based on our results, we estimate that these primers are suitable for studying a large number of nuclear genes across a wide range of apocritan Hymenoptera (i.e., all hymenopterans with a wasp-waist) and of aculeate Hymenoptera in particular (i.e., apocritan wasps with stingers).

**Conclusions:**

The amplified nucleotide sequences are (a) with high probability from single-copy genes, (b) easily generated at low financial costs, especially when compared to phylogenomic approaches, (c) easily sequenced by means of an additionally provided set of sequencing primers, and (d) suitable to address a wide range of phylogenetic questions and to aid rapid species identification via barcoding, as many amplicons contain both exonic and fast-evolving intronic nucleotides.

## Introduction

Targeted amplification of single-copy genes is still a cornerstone of molecular phylogenetics despite the emergence of phylogenomic approaches analyzing transcriptome data and entire genomes. PCR approaches have focused primarily on mitochondrial genes, rRNA, and a restricted number of nuclear genes [1]–[4]. The phylogenetic analysis of a set of these standard genes and the study of phylogenomic data both have their pros and cons: the few standard genes are comparatively easy to amplify across a wide range of species, but their phylogenetic signal may be insufficient to answer the research question(s) of interest. In contrast, phylogenomic approaches provide a plethora of nucleotide sequence data and facilitate addressing difficult phylogenetic questions. However, phylogenomic approaches are (still) expensive and may require specially treated sample material (e.g., for preservation of RNA), which means that material from most scientific collections cannot be used. Degenerate oligonucleotide PCR primers designed to amplify a large set of single-copy nuclear genes in species of interest could close the gap between the two approaches and could be a viable alternative to both of them. Here, we present such a suite of PCR primers for amplifying single-copy nuclear genes from Hymenoptera (sawflies, bees, wasps, and ants).

Hymenoptera are one of the mega-diverse insect orders and encompass more than 125,000 described species, many of which have key functions in ecosystems and are of fundamental economical, agricultural, and medical importance [5]. Given this importance, it is surprising how few molecular markers are currently in use for phylogenetic and evolutionary studies of Hymenoptera (e.g., [6]–[9]). Even the most recent comprehensive phylogenetic investigation of Hymenoptera used a PCR approach that targeted only four genes (18S, 28S, EF1α, COX1) [4]. Many important nodes in the resulting phylogeny are not robust, indicating that more nucleotide sequence data are required to answer these and other fundamental phylogenetic questions involving Hymenoptera. Additionally, only two phylogenomic studies have been published that analyze EST data from Hymenoptera, both with very limited taxon samples [10], [11]. Peters and colleagues [12] combined all published sequence data of Hymenoptera for a comprehensive phylogenetic analysis. This study revealed that only about ten molecular markers are frequently used to tackle phylogenetic questions in the Hymenoptera. These markers have undoubtedly given important insights into the evolutionary history of this group. Nonetheless, their limited phylogenetic signal has also left many difficult and longstanding phylogenetic questions unresolved.

Genome sequence data offer new opportunities to establish markers for phylogenetic and evolutionary studies. This strategy has already successfully been pursued for fungi [13]. In Hymenoptera, nine genomes have been published (seven ants [14]–[19]; honeybee [20]; parasitoid wasp [21]). These genomes offer a rich and unexploited library of molecular markers for phylogenetic analyses.

There are three major advantages of establishing molecular markers for phylogenetic analyses from sequenced genomes compared to traditional approaches and to the exploration of EST data: (a) the ability to reliably assess the orthology of genes; (b) the ability to assess the probability of obtaining undesired secondary PCR products; and (c) the availability of gene models that inform about the position and length of introns and exons. One-to-one orthologous (single-copy) protein-coding genes can be identified with high confidence using orthology assessment software such as OrthoMCL [22]. When restricting oligonucleotide primer design to single-copy genes, the risk of accidentally sequencing pseudogenes and other paralogous genes is greatly reduced. If the main interest of a study lies in amplifying fast evolving sites, for example to address relationships within species or among closely related species, it is possible to focus on PCR primer pairs that maximize the amount of intronic sites in the PCR product. This kind of information cannot be inferred from EST data.

We present a suite of new degenerate oligonucleotide primers that are expected to amplify single-copy nuclear protein-coding genes in a taxonomically wide array of apocritan Hymenoptera (i.e., Hymenoptera with a wasp-waist). This lineage of Hymenoptera comprises the vast majority (>95%) of hymenopteran species [5]. We provide detailed primer statistics and a PCR protocol for rapidly assessing the functionality of primer pairs, and we show results from empirically testing ten randomly selected primer pairs on DNA from six Hymenoptera species, representing the families Chrysididae, Crabronidae, Gasteruptiidae, Leucospidae, Pompilidae, and Stephanidae. The targeted molecular markers can be used to address a wide range of phylogenetic and/or comparative evolutionary questions, may prove valuable for rapid species identification via barcoding, and can be easily generated at low financial costs.

## Methods

### Search for and Annotation of 1∶1 Orthologous Genes

We searched for orthologous genes in the genomes of nine Hymenoptera: a parasitoid wasp (*Nasonia vitripennis*; Pteromalidae; assembly 1.0; OGS 1.2) [21], the honeybee (*Apis mellifera*; Apidae; assembly 2.0; OGS pre-release 2) [20], Jerdon’s jumping ant (*Harpegnathos saltator*; Formicidae: Ponerinae; assembly 3.3; OGS 3.3) [14], the Argentine ant (*Linepithema humile*; Dolichoderinae; assembly 1.0; OGS 1.1) [15], the Florida carpenter ant (*Camponotus floridanus*; Formicinae; assembly 3.3; OGS 3.3) [14], the red harvester ant (*Pogonomyrmex barbatus*; Myrmicinae; assembly 3.0; OGS 1.1) [16], the red fire ant (*Solenopsis invicta*; Myrmicinae; assembly 1.0; OGS 2.2) [17], and two leaf-cutter ants (*Atta cephalotes*; Myrmicinae; assembly 4.0; OGS 1.1; *Acromyrmex echinatior*; Myrmicinae; assembly 1.0; OGS 1.0) [18], [19].

Orthology of proteins between the nine genomes was inferred using a graph-based approach as implemented in OrthoMCL 2.0 [22]. This approach has been shown to have reasonably low false positive and false negative rates among the available methods to estimate gene orthology [23]. We only used sequence pairs from the ‘orthologs.txt’ output file for Markov clustering. The inflation value was set to 1.5. Finally, we extracted sets of 1∶1 orthologs from the final OrthoMCL output file with the aid of a custom-made Perl script. The amino acids in each set of 1∶1 orthologous proteins were aligned with MAFFT 6.833b [24], [25] using the ‘L-INS-I’ alignment strategy. Note that we replaced the amino acid code ‘U’, which stands for selenocysteine and is not recognized by MAFFT, with the ambiguity code ‘X’ prior to alignment. The alignment was subsequently refined with MUSCLE 3.7 [26] using the refinement option. Each amino acid alignment was then used as a blueprint to align the nucleotides of the corresponding coding sequences with a custom-made Perl script and the BioPerl tool kit [27]. All sets of 1∶1 orthologs were annotated by generating profile hidden Markov models (pHMMs) from the protein alignments. The pHMMs were used to search the official gene set (OGS) of the fruit fly *Drosophila melanogaster* (FlyBase release 5.22) [28] for the most similar sequence (*E* value <10^−10^) with the HMMER 3.0 [29], [30] software package. We also estimated the average nucleotide sequence divergence among the nine reference genomes for each amplified region by calculating Hamming distances ( =  uncorrected p-distances) using a custom-made Perl script.

### Oligonucleotide Primer Design

All 4,145 multiple nucleotide alignments of 1∶1 orthologous genes were searched for suitable primer binding sites using a custom-made Ruby script (Janus Borner, Christian Pick, Thorsten Burmester, unpublished). The script designs degenerate primers for PCR-amplification of coding sequences from the nuclear genome. It searches for conserved regions in aligned protein-coding nucleotide sequences and checks whether or not possible oligonucleotide primers that would bind at these conserved regions do not exceed a certain degree of degeneration, exhibit a GC content within a given range, and do not possess more than a given number of nucleotide repeats (Table 1). All primer pairs consistent with these criteria were searched for matches in the genomic nucleotide sequences of the nine reference species. This allowed estimating the actual length and the relative intron content of each amplicon. Primers that did not match because they bind at an exon/intron boundary or because they would amplify a region exceeding a pre-defined size (Table 1), were discarded. Approximate genomic matches were also considered to assess the probability of obtaining undesired secondary amplification products. To allow for direct sequencing of the PCR products using specific oligonucleotide sequencing primers, pre-designed oligonucleotides were added to the 5′ end of each primer sequence (Table 2). Finally, we evaluated the melting temperatures and hybridization energies of homo- and heterodimers for each pair of primers with the aid of UNAFold 3.8 [31]. All primer design parameters are summarized in Table 1.

**Table 1:** Oligonucleotide PCR primer design parameters.

Parameter	Minimum Value	Maximum Value
Amplicon length (bp)	300	1000
Primer length (bp)	20	25
Degree of degeneration	–	256
GC content (%)	20	80
Repeats of single nucleotide (bp)	–	4
Melting temperature (°C)	45	66
Difference of melting temperatures (°C)	–	10
dG of homodimer (kcal/mole)	−11.0	–
dG of heterodimer (kcal/mole)	−11.0	–
Degree of degeneration at 3′ end*	–	4
GC content (%) at 3′ end*	20	80
Repeats of single nucleotide (bp) at 3′ end*	–	3

* Terminal six nucleotides.

### Empirical Evaluation of Oligonucleotide Primer Pairs

**Figure 1:**
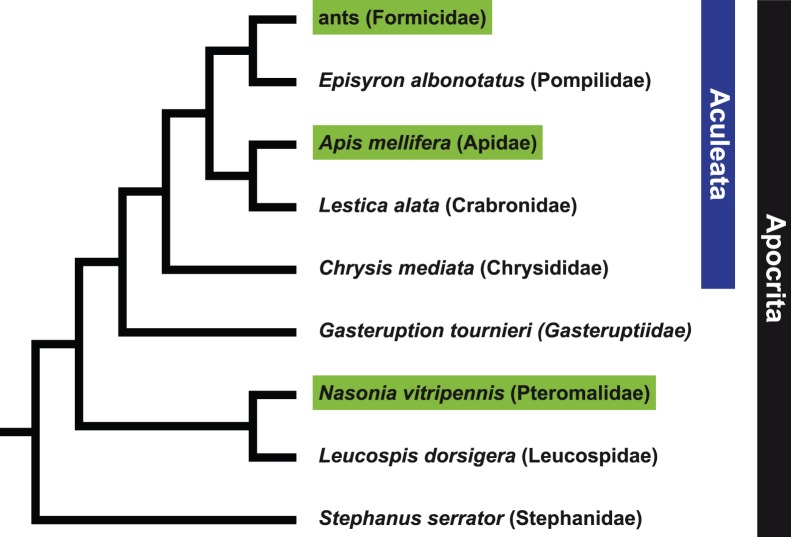
Hypothesized phylogenetic relationships of apocritan Hymenoptera studied in this investigation [4], [12]. Taxa with sequenced genomes are highlighted in green; their genome sequences were analyzed to identify single-copy genes and to design degenerate oligonucleotide PCR primers. DNA of non-highlighted species was used to assess the functionality of the inferred PCR and sequencing primers.

Ten randomly chosen PCR primer pairs, each with the forward and reverse oligonucleotide primers of sequencing primer set HOG-Seq A (Table 2) attached to their 5′ ends, were tested for amplifying the target genes in six apocritan Hymenoptera: *Stephanus serrator* (Stephanidae), *Leucospis dorsigera* (Leucospidae), *Gasteruption tournieri* (Gasteruptiidae), *Chrysis mediata* (Chrysididae), *Lestica alata* (Crabronidae), and *Episyron albonotatum* (Pompilidae). With Stephanidae, the possible sister group of all remaining Apocrita, and with representatives of the superfamilies Chalcidoidea (*Leucospis*), Evanioidea (*Gasteruption*), Chrysidoidea (*Chrysis*), Apoidea (*Lestica*), and Vespoidea (*Episyron*), our taxon sampling includes representatives of several deeply-divergent major lineages of the mega-diverse Hymenoptera (Figure 1). All taxa were collected by ON in Rhineland-Palatinate, Germany, in 2011 and were preserved in 96% ethanol.

**Table 2:** Oligonucleotide sequencing primer pairs.

Primer pair	Forward (5′ → 3′)	*T*_m_	Reverse (5′ → 3′)	*T*_m_
**HOG-Seq-A**	CAGTAGGTGCGTATGTCA	49.9	TGGTCAGTGGCTATTCGT	50.9
**HOG-Seq-B**	CGCTCATACACTTGGTTC	49.7	TCAGTCATCCTCACTTCG	50.3
**HOG-Seq-C**	ATACTAACTGGTGGAGCGAG	52.6	TCACTACATTACCGTATGAC	48.6
**HOG-Seq-D**	TCGGTCACATTGGGCTACT	54.5	CCTTGGGTCTTCGGCTTGA	56.5

The nucleotide sequences of the sequencing primers were attached as a binding site to the 5′ end of the degenerate oligonucleotide polymerase chain reaction (PCR) primers. Each of the oligonucleotide primers in Table S1 is compatible with at least one of the sequencing primers added to the 5′ end of the PCR primer. *T*_m_  =  approximate melting temperature [°C].

DNA was extracted from thoracic muscle tissue using the QIAGEN DNeasy Blood & Tissue Kit and following the protocol for insects (QIAGEN GmbH, Hilden, Germany). DNA quality and quantity were assessed by running the extracted DNA on a 1.5% agarose gel and by analyzing the DNA with a NanoDrop 1000 Spectrophotometer (NanoDrop Technologies, Wilmington, DE, USA). Polymerase chain reactions (PCRs) were run in 20 µl volumes consisting of 0.5× QIAGEN Q-Solution, 1× QIAGEN Multiplex PCR Master Mix (QIAGEN GmbH, Hilden, Germany), 0.8 µM of each oligonucleotide primer, and 50 ng DNA. The touch-down PCR temperature profile started with an initial denaturation and QIAGEN HotStarTaq DNA polymerase activation step at 95°C for 15 min., followed by 16 cycles of 95°C for 0.5 min., 60–45°C for 0.5 min., and 72°C for 1.5 min, followed by 20 cycles of 95°C for 0.5 min., 65 for 0.5 min., and 72°C for 1.5 min, followed by 10 min. at 72°C. Note that the annealing temperature (*T*_a_) was decreased during the first 16 cycles by 1°C each cycle. All PCRs were run on a Biometra Whatman T3000 Thermocycler (Biometra GmbH, Göttingen, Germany). PCR products were separated on a 1.5% agarose gel, together with a Fermentas 100 bp Plus DNA Ladder (Fermentas GmbH, Sankt Leon-Rot, Germany). PCR products chosen for sequencing (i.e., the amplicons of the five best-performing PCR primer pairs) were purified with the QIAquick PCR Purification Kit (QIAGEN GmbH, Hilden, Germany) and sent to Macrogen Inc. (Amsterdam, Netherlands) for direct Sanger sequencing with the sequencing primers HOG-Seq-A-F and HOG-Seq-A-R (Table 2). Forward and reverse DNA strands were assembled to contigs, trimmed (to exclude the binding sites of the PCR and sequencing oligonucleotide primers), and aligned with Geneious Pro 5.4.6 [32] to the sequences of the nine Hymenoptera, from which the primer pairs were inferred.

**Figure 2:**
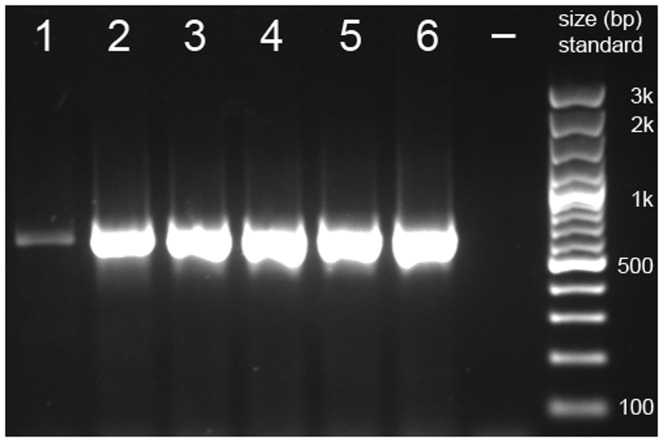
Polymerase chain reaction (PCR) products separated on 1.5% agarose gel. The depicted gel shows the PCR products obtained from using the inferred oligonucleotide primer pair 7229_02_A (Table 3) to PCR amplify DNA of *Stephanus serrator* (Stephanidae, 1), *Leucospis dorsigera* (Leucospidae, 2), *Gasteruption tournieri* (Gasteruptiidae, 3), *Chrysis mediata* (Chrysididae, 4), *Lestica alata* (Crabronidae, 5), and *Episyron albonotatum* (Pompilidae, 6). All PCR products were suitable for direct sequencing with the sequencing oligonucleotide primers HOG-Seq-A-F/−R (Table 2). –  =  negative control.

All new sequences generated in this study have been submitted to the European Nucleotide Archive (accession numbers HE612159–HE612181).

**Table 3:** Empirically evaluated degenerate oligonucleotide PCR primer pairs.

ID	Forward (5′ → 3′)	*d*	*T*_m_ min	*T*_m_ max	Reverse (5′ → 3′)	*d*	*T*_m_ min	*T*_m_ max	Total *d*
**3683_01_A**	GCYATYTTCGAYTTYGAYAG	32	46.0	56.8	AAVGTRAAKGATTCGTTGTA	12	47.5	54.4	384
**4652_02_A**	ATGATGTDGARTTTATMATACARAC	24	46.9	53.8	CWACRCTWATTTCTCTWTCAAC	16	47.1	51.9	384
**4747_02_A**	TTCTACGGBATGATCTTYAG	6	47.1	53.0	ACCTBGACATRATCTTVGGC	18	49.8	57.2	108
**5119_01_A**	GGDATYGTMGARGAGAGYGT	48	48.7	60.8	TYTTCATYTTRTCCATGTGYTC	16	48.9	56.5	768
**5257_01_A**	MACVAATAARTAYGGHTGYAGA	144	46.7	58.9	TAATTGGTCTARRTTGAARCT	8	47.0	52.7	1,152
**5592_01_A**	AAYTRAATAAAGACTGGAAAGAAGA	4	50.3	53.8	GTYARATCCATYCCRTGATC	16	47.6	55.7	64
**5768_01_A**	ACDGTHAARGTDTGGAATGC	54	48.6	58.2	GCWACCCAAATRCWAGWTTG	16	48.8	55.0	864
**6917_01_A**	ATGCCVTTCTACACRGTCTA	6	52.8	58.0	CYTCGCTYTTCTTCTGCATRTC	8	53.5	58.9	48
**7036_02_A**	TTTGTCWGYGKGTGCCTTGT	8	55.4	60.1	TTCATRGTWGCTTCRGTATCNGT	32	51.2	59.2	256
**7229_02_A**	TGCYTGATHCTSTTCTTCGT	12	51.1	55.8	TRTGRAAYCTRTGRAAGATGCA	32	49.6	58.6	384

The ten degenerate oligonucleotide primers were tested with the respective binding sites for sequencing primer HOG-Seq-A (see Table 2) attached to the 5′ end and used to amplify ten target genes in six apocritan Hymenoptera. *d*  =  degree of degeneration. *T*_m_  =  approximate melting temperature [°C].

**Table 4:** Rating of obtained polymerase chain reaction (PCR) products.

Marker	*S. serrator*	*L. dorsigera*	*G. tournieri*	*C. mediata*	*L. alata*	*E. albonotatum*
**3683_01_A**	++*	+/−	++	++	++	++
**4652_02_A**	–	–	–	–	–	+
**4747_02_A**	–	++	+	++	+*	++
**5119_01_A**	–	++* (?)	–	++* (?)	++*	++*
**5257_01_A**	–	–	–	–	++	++
**5592_01_A**	–	++	+/−	++	++	++
**5768_01_A**	–	–	–	+/−*	+	–
**6917_01_A**	–	++	+	++	++	++
**7036_02_A**	+/−	++	++	++	++	++
**7229_02_A**	+	++	++	++	++	++

Rating of the PCR products obtained from using the degenerate oligonucleotide primers shown in Table 3 to amplify ten target genes in six apocritan Hymenoptera. ++  =  target PCR product in excess. +  =  target PCR product sufficient for direct sequencing. +/−  =  target PCR product insufficient for direct sequencing. –  =  no target PCR product. (?)  =  unclear whether or not PCR products include amplicon of target gene.

* Secondary PCR amplification product likely hampering direct sequencing.

**Table 5:** European Nucleotide Archive accession numbers for all sequences generated from primer testing.

Marker	*S. serrator*	*L. dorsigera*	*G. tournieri*	*C. mediata*	*L. alata*	*E. albonotatum*
**3683_01_A**	failed	NA	HE612159	HE612169	HE612174	HE612181
**5592_01_A**	NA	HE612164	NA	HE612170	HE612175	HE612180
**6917_01_A**	NA	HE612165	HE612167	HE612168	HE612177	failed
**7036_02_A**	NA	HE612166	HE612160	HE612171	HE612176	HE612179
**7229_02_A**	HE612162	HE612163	HE612161	HE612172	HE612173	HE612178

## Results

### Gene Orthology and Oligonucleotide Primer Design

Analyzing the official gene sets of the nine hymenopterans with sequenced genomes, we identified a total of 4,145 single-copy orthologous genes that were present in every species. Studying the multiple nucleotide sequence alignments of these 4,145 orthologous genes, we inferred 304 oligonucleotide primer pairs for amplifying a total of 154 single-copy nuclear protein-coding genes. The length of the inferred primers ranges between 20 and 25 nucleotides (avg. 21), their estimated *T*_m_ (approximate melting temperature) ranges between 44.6° and 65.7°C (avg. 53.5°C), and their degree of degeneration ranges between 1 and 192 (avg. 31). For each of the 154 genes, we inferred between 1 and 11 (avg. 2) primer pairs with a maximum overlap between amplicons of 50%. The total degree of degeneration of the primer pairs ranges between 2 and 12,288 (avg. 860) (Table S1). The expected sizes of the PCR products range between 378 and 1,074****bp (avg. 683 bp; N = 2,736), the expected sizes of the amplified target regions range between 301 and 996 bp (avg. 604 bp; N = 2,736), and the average uncorrected (p) distance among the amplified target regions in the nine reference genomes ranges between 7.9% and 35.3% (avg. 17.3%; N = 304).

Of the 304 inferred oligonucleotide primer pairs, 233 amplify genomic regions that according to the available gene models include at least one predicted intron in the reference genomes. These primer pairs amplify 112 (∼73%) of the 154 genes. In contrast, only 28 inferred oligonucleotide primer pairs amplify genomic regions that do not include introns in the nine reference species. These primer pairs amplify 17 different (∼11% of the here covered 154) genes. All remaining primer pairs amplify genomic regions that may or may not include introns. The number of exonic nucleotides in those 233 genomic target regions that include at least one predicted intron ranges from 154 to 814 (avg. 429; N = 2,097). The corresponding number of intronic nucleotides ranges from 45 to 653 (avg. 195; N = 2,097). The percentage of exonic nucleotides in the above mentioned 233 genomic regions ranges from 22.4 to 93.4 (avg. 69.8; N = 2,097). The number of exonic nucleotides in those 28 genomic regions that do not contain introns in any of the reference species ranges from 304 to 816 (avg. 454; N = 252).

Of the 304 inferred oligonucleotide primer pairs, we found 80 (referring to 71 different genes) to be compatible with sequencing primer pair HOG-Seq-A, 130 (referring to 107 different genes) to be compatible with sequencing primer pair HOG-Seq-B, 46 (referring to 46 different genes) to be compatible with sequencing primer pair HOG-Seq-C, and 73 (referring to 62 different genes) to be compatible with sequencing primer pair HOG-Seq-D (Table 2).

The complete list of inferred degenerate oligonucleotide primers, along with complementary information (e.g., annealing temperature, degree of degeneration, expected length of amplicons, compatibility with sequencing primers attached to the 5′ end of the PCR primers) is given in Table S1. Additional supplementary material has been deposited in the Dryad data repository (http://datadryad.org/; doi:10.5061/dryad.d73k0).

### Empirical Evaluation of Oligonucleotide Primer Pairs

All tested PCR primers had the oligonucleotides of the sequencing primer pair set HOG-Seq A attached to their 5′ ends (Table 2 and Table 3). One pair of tested primers produced amplicons suitable for direct sequencing in all six species (Tables 4 and 5, Figure 2). An additional five primer pairs produced amplicons suitable for direct sequencing in at least four of the six studied species, with a tendency to less reliably produce a PCR product suitable for direct sequencing with increasing evolutionary distance from ants (Tables 4 and 5, Figure S1). Overall, the PCR success rate when using DNA from species of Aculeata (i.e., apocritan wasps with stingers) was ∼80%. When considering all Apocrita, the PCR success rate was still ∼60%.

## Discussion

We inferred 304 oligonucleotide primer pairs that can be used for PCR amplification of up to 154 different genes in apocritan Hymenoptera. The ten primer pairs that were empirically tested proved to be highly successful in amplifying the desired target DNA of Aculeata and showed a reasonable success-rate when applied to DNA of other Apocrita. Extrapolating these results and considering that we provide on average two primer pairs for a given gene, we expect up to 148 genes of interest to be amplifiable in aculeate Hymenoptera and roughly 110 to be amplifiable in many other groups of Apocrita. The high success-rate of our new PCR primers is most likely the result of the strict selection criteria that we applied during primer design (e.g., low potential for self-priming and the formation of hairpin loops, no alternative binding sites in the reference genomes). However, given that seven of the nine analyzed reference genomes are from ants, we expect fewer primers to amplify the desired product when they are applied to DNA of species that are distantly related to ants (e.g., non-aculeate Apocrita).

There are several options for improving the PCR success-rate of the primers reported here. For example, while we used a touch-down temperature profile to rapidly assess the functionality of the ten evaluated primer pairs, one could instead use a PCR temperature profile with a constant annealing temperature that is close to the optimal annealing temperature of the specific primer pair (Table S1). Such a temperature profile could reduce the risk of obtaining secondary amplification products. Since we did not apply primer-specific PCR temperature profiles when empirically testing primer pairs, we expect their success-rate to be slightly underestimated. Researchers using these new primers should also consider increasing the concentration of oligonucleotides in the PCR mix to counterbalance the high degree of degeneration of some of the oligonucleotides (Table S1).

We calculated the average nucleotide sequence divergence among the nine reference genomes for the amplified region plus the absolute number of intronic and exonic nucleotides in the expected amplicon for each primer pair (Table S1). Consequently, users are able to search for markers that are more- or less-conserved than others, and users are additionally able to select for primers that specifically amplify genes with or without introns. Intronic DNA could prove highly valuable for resolving genealogical relationships of recently diverged lineages. These nuclear markers may also prove to be very useful for DNA barcoding. Overall, the ability to select genes that seem particularly suitable to address a specific research question makes the plethora of PCR primers presented here a highly valuable toolbox for research in apocritan Hymenoptera. Finally, the inferred primers are compatible with pre-designed oligonucleotides (Table 2) attached to their 5′ end. This allows users to select a single oligonucleotide sequencing primer pair from a set of four for sequencing all PCR products.

Our approach for designing oligonucleotides for PCR-amplification of orthologous genes in a wide range of species requires the availability of sequenced genomes. One group of insects, besides Hymenoptera, for which genomes of several taxa have been sequenced, and for which such an approach might prove fruitful, is Diptera. Genome sequences from more than 15 species of Diptera are currently available and those of many more are already in progress. As in Hymenoptera, however, there is a strong taxonomic bias: only genomes of fruit flies (*Drosophila* spp.) and of mosquitos (Culicidae) have been published. As these two taxa belong to two distantly related lineages that split early in the evolution of Diptera, the available genomes might nonetheless already reflect a significant proportion of the molecular diversity in Diptera. With the i5K initiative [33], we expect the number of sequenced insect genomes to explode in the very near future. This will likely allow the inference of large numbers of phylogenetic markers for many more insect orders.

## Supporting Information

Figure S1 **Polymerase chain reaction (PCR) products separated on 1.5% agarose gels.** The depicted gels show the PCR products obtained from using the inferred oligonucleotide primer pairs **A.** 3683_01_A, **B.** 4652_02_A, **C.** 4747_02_A, **D.** 5119_01_A, **E.** 5257_01_A, **F.** 5592_01_A, **G.** 5768_01_A, **H.** 6917_01_A, and **I.** 7036_02_A (see Table 3) to PCR amplify DNA of **1.** Stephanus serrator (Stephanidae), **2.** Leucospis dorsigera (Leucospidae), **3.** Gasteruption tournieri (Gasteruptiidae), **4.** Chrysis mediata (Chrysididae), **5.** Lestica alata (Crabronidae), and **6.** Episyron albonotatum (Pompilidae). –  =  negative control. L  = 100 bp ladder (see also Figure 2).
(TIF)

Table S1 **Inferred degenerate oligonucleotide primers for studying single-copy nuclear genes in apocritan Hymenoptera.**
(XLS)

## References

[1] Brower AVZ, DeSalle R (1994). Practical and theoretical considerations for choice of a DNA sequence region in insect molecular systematics, with a short review of published studies using nuclear gene regions. Ann. Entomol. Soc. Am..

[2] Simon C, Buckley TR, Frati F, Stewart JB, Beckenbach AT (2006). Incorporating molecular evolution into phylogenetic analysis, and a new compilation of conserved polymerase chain reaction primers for animal mitochondrial DNA. Annu. Rev. Ecol. Evol. Syst.. http://dx.doi.org/10.1146/annurev.ecolsys.37.091305.110018.

[3] Wiegmann BM, Trautwein MD, Kim J-W, Cassel BK, Bertone MA (2009). Single-copy nuclear genes resolve the phylogeny of the holometabolous insects. BMC Biol.. http://dx.doi.org/10.1186/1741-7007-7-34.

[4] Heraty J, Ronquist F, Carpenter JM, Hawks D, Schulmeister S (2011). Evolution of the hymenopteran megaradiation. Mol. Phylogenet. Evol.. http://dx.doi.org/10.1016/j.ympev.2011.04.003.

[5] Grimaldi D, Engel MS (2005). Evolution of the insects. New York, NY: Cambridge University Press.. 755 pp.

[6] Dowton M, Austin AD (1994). Molecular phylogeny of the insect order Hymenoptera: apocritan relationships. Proc. Natl. Acad. Sci. U.S.A..

[7] Dowton M, Austin AD (2001). Simultaneous analysis of 16S, 28S, COI and morphology in the Hymenoptera: Apocrita – evolutionary transitions among parasitic wasps. Biol. J. Linnean Soc.. http://dx.doi.org/l0.1006/bij1.2001.0577.

[8] Desjardins CA, Regier JC, Mitter C (2007). Phylogeny of pteromalid parasitic wasps (Hymenoptera: Pteromalidae): initial evidence from four protein-coding nuclear genes. Mol. Phylogenet. Evol.. http://dx.doi.org/10.1016/j.ympev.2007.08.004.

[9] Pilgrim EM, Von Dohlen CD, Pitts JP (2008). Molecular phylogenetics of Vespoidea indicate paraphyly of the superfamily and novel relationships of its component families and subfamilies. Zool.. Scripta.

[10] Sharanowski BJ, Robbertse B, Walker J, Voss SR, Yoder R (2010). Expressed sequence tags reveal Proctotrupomorpha (minus Chalcidoidea) as sister to Aculeata (Hymenoptera: Insecta). Mol. Phylogenet. Evol.. http://dx.doi.org/10.1016/j.ympev.2010.07.006.

[11] Woodard SH, Fischman BJ, Venkat A, Hudson ME, Varala K (2011). Genes involved in convergent evolution of eusociality in bees. Proc. Natl. Acad. Sci. U.S.A.. http://dx.doi.org/10.1073/pnas.1103457108.

[12] Peters RS, Meyer B, Krogmann L, Borner J, Meusemann K (2011). The taming of an impossible child: a standardized all-in approach to the phylogeny of Hymenoptera using public database sequences. BMC Biol.. http://dx.doi.org/10.1186/1741-7007-9-55.

[13] Feau N, Decourcelle T, Husson C, Desprez-Loustau M-L, Dutech C (2011). Finding single copy genes out of sequenced genomes for multilocus phylogenetics in non-model fungi.. PLoS ONE.

[14] Bonasio R, Zhang G, Ye C, Mutti NS, Fang X (2010). Genomic comparison of the ants *Camponotus floridanus* and *Harpegnathos saltator*.. Science.

[15] Smith CD, Zimin A, Holt C, Abouheif E, Benton R (2011). Draft genome of the globally widespread and invasive Argentine ant (*Linepithema humile*). Proc. Natl. Acad. Sci. U.S.A.. http://dx.doi.org/10.1073/pnas.1008617108.

[16] Smith CR, Smith CD, Robertson HM, Helmkampf M, Zimin A (2011). Draft genome of the red harvester ant *Pogonomyrmex barbatus*. Proc. Natl. Acad. Sci. U.S.A.. http://dx.doi.org/10.1073/pnas.1007901108.

[17] Wurm Y, Wang J, Riba-Grognuz O, Corona M, Nygaard S (2011). The genome of the fire ant *Solenopsis invicta*. Proc. Natl. Acad. Sci. U.S.A.. http://dx.doi.org/10.1073/pnas.1009690108.

[18] Nygaard S, Zhang G, Schiøtt M, Li C, Wurm Y (2011). The genome of the leaf-cutting ant *Acromyrmex echinatior* suggests key adaptations to advanced social life and fungus farming. Genome Res.. http://dx.doi.org/10.1101/gr.121392.111.

[19] Suen G, Teiling C, Li L, Holt C, Abouheif E (2011). The genome sequence of the leaf-cutter ant *Atta cephalotes* reveals insights into its obligate symbiotic lifestyle. PLoS Genet.. http://dx.doi.org/10.1371/journal.pgen.1002007.

[20] Weinstock GM, Robinson GE, Gibbs RA, Worley KC, Evans JD (2006). Insights into social insects from the genome of the honeybee *Apis mellifera*.. Nature.

[21] Werren JH, Richards S, Desjardins CA, Niehuis O, Gadau J (2010). Functional and evolutionary insights from the genomes of three parasitoid *Nasonia* species.. Science.

[22] Li L, Stoeckert CJ, Roos DS (2003). OrthoMCL: Identification of ortholog groups for eukaryotic genomes. Genome Res.. http://dx.doi.org/10.1101/gr.1224503.

[23] Chen F, Mackey AJ, Vermunt JK, Roos DS (2007). Assessing performance of orthology detection strategies applied to eukaryotic genomes.. PLoS ONE.

[24] Katoh K, Kuma K, Toh H, Miyata T (2005). MAFFT version 5: improvement in accuracy of multiple sequence alignment. Nucleic Acids Res.. http://dx.doi.org/10.1093/nar/gki198.

[25] Katoh K, Toh H (2008). Recent developments in the MAFFT multiple sequence alignment program. Brief.. Bioinformatics.

[26] Edgar RC (2004). MUSCLE: a multiple sequence alignment method with reduced time and space complexity.. BMC Bioinformatics.

[27] Stajich JE, Block D, Boulez K, Brenner SE, Chervitz SA (2002). The Bioperl toolkit: Perl modules for the life sciences. Genome Res.. http://dx.doi.org/10.1101/gr.361602.

[28] Adams MD, Celniker SE, Holt RA, Evans CA, Gocayne JD (2000). The genome sequence of *Drosophila melanogaster*.. Science.

[29] Eddy SR (1998). Profile hidden Markov models.. Bioinformatics.

[30] Finn RD, Clements J, Eddy SR (2011). HMMER web server: interactive sequence similarity searching. Nucleic Acids Res.. http://dx.doi.org/10.1093/nar/gkr367.

[31] Markham NR, Zuker M (2008). UNAFold: software for nucleic acid folding and hybridization. Methods Mol. Biol.. http://dx.doi.org/10.1007/978-1-60327-429-6_1.

[32] Drummond AJ, Ashton B, Buxton S, Cheung M, Cooper A (2011). Geneious. Auckland (New Zealand): Biomatters Ltd.. p.

[33] Robinson GE, Hackett KJ, Purcell-Miramontes M, Brown SJ, Evans JD (2011). Creating a buzz about insect genomes.. Science.

