# Supplementary figures and images for: Oligonucleotide Primers for Targeted Amplification of Single-Copy Nuclear Genes in Apocritan Hymenoptera
Source: PLoS One. 2012 Jun 29;7(6):e39826. doi: 10.1371/journal.pone.0039826 (PMC3387199; doi:10.1371/journal.pone.0039826)

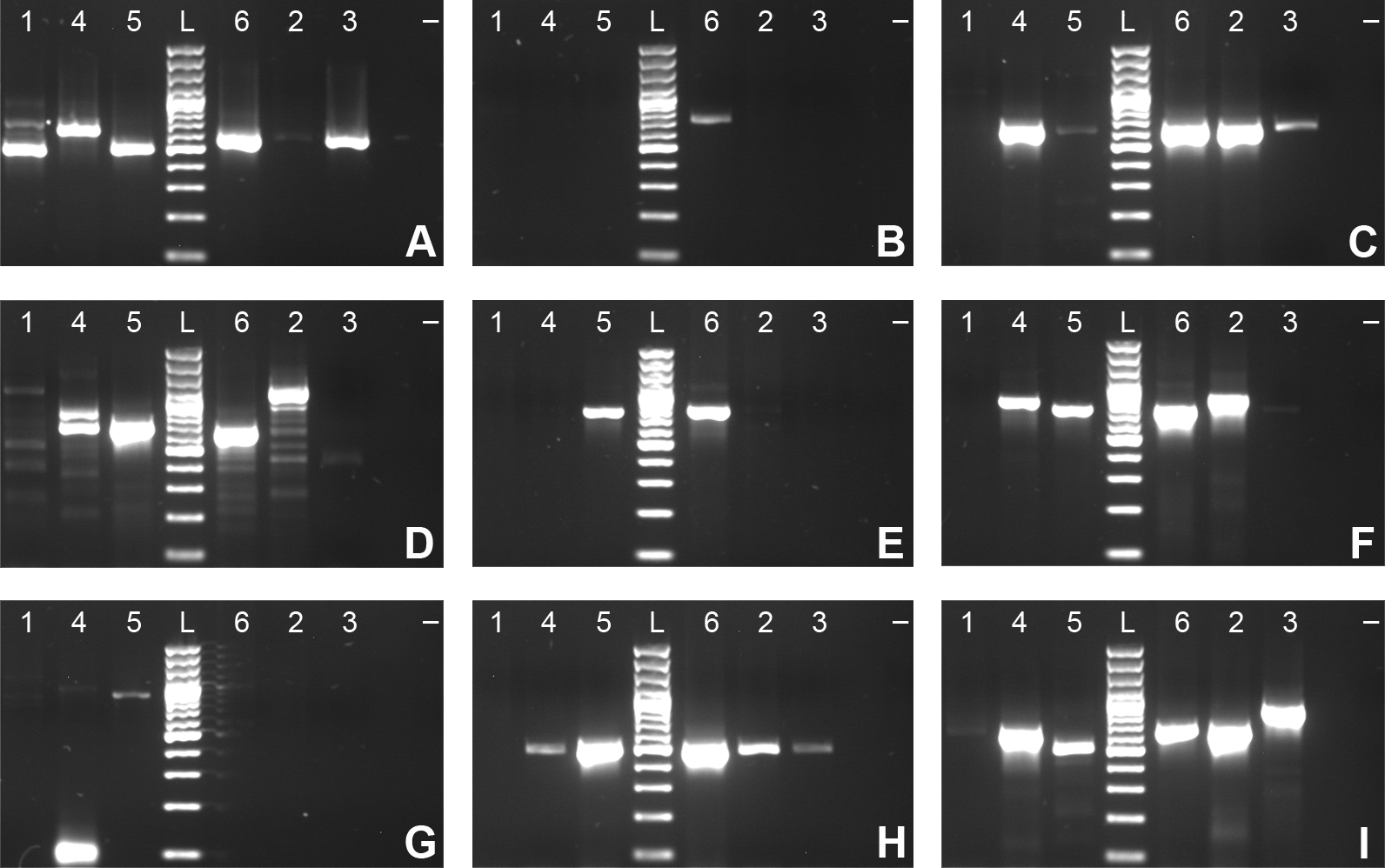

Supplement: Figure S1 — Polymerase chain reaction (PCR) products separated on 1.5% agarose gels. The depicted gels show the PCR products obtained from using the inferred oligonucleotide primer pairs A. 3683_01_A, B. 4652_02_A, C. 4747_02_A, D. 5119_01_A, E. 5257_01_A, F. 5592_01_A, G. 5768_01_A, H. 6917_01_A, and I. 7036_02_A (see Table 3) to PCR amplify DNA of 1. Stephanus serrator (Stephanidae), 2. Leucospis dorsigera (Leucospidae), 3. Gasteruption tournieri (Gasteruptiidae), 4. Chrysis mediata (Chrysididae), 5. Lestica alata (Crabronidae), and 6. Episyron albonotatum (Pompilidae). – = negative control. L = 100 bp ladder (see also Figure 2). (TIF) [file pone.0039826.s001.tif]
